# Ferulic Acid Protects against Porcine Parvovirus Infection-Induced Apoptosis by Suppressing the Nuclear Factor-*κ*B Inflammasome Axis and Toll-Like Receptor 4 via Nonstructural Protein 1

**DOI:** 10.1155/2020/3943672

**Published:** 2020-04-25

**Authors:** Xia Ma, Zhenhuan Guo, Zhiqiang Zhang, Xianghui Li, Yonglu Liu, Li Zhao, Xuefei Wang

**Affiliations:** ^1^College of Pharmaceutical Engineering, Henan University of Animal Husbandry and Economy, Zhengzhou 450046, China; ^2^Department of Pharmacology, School of Pharmacy, Nanjing University of Chinese Medicine, Nanjing 210023, China

## Abstract

**Background:**

Porcine parvovirus (PPV) infection-induced apoptosis was recently identified as an important pathological factor in PPV-induced placental tissue damage, resulting in reproduction failure. In the present study, we demonstrate the possible involvement of toll-like receptor (TLR) 4 and nuclear factor (NF)-*κ*B inflammasome activation in PPV infection-induced apoptosis and the protective potential of ferulic acid (FA). PPV infection significantly activated the expression levels of TLR4, NF-*κ*B, MyD88, and interleukin (IL)-6. However, FA ameliorated the pathological process, prevented histological alterations, and inhibited the apoptosis rate in porcine kidney (PK-15) cells infected with PPV.

**Results:**

FA inhibited PPV infection-induced inflammasome activation as shown by decreases in the expression of NF-*κ*B, MyD88, and IL-6. FA also downregulated nonstructural (NS) 1 protein expression in infected PK-15 cells.

**Conclusions:**

FA downregulated NS1 and TLR4 signaling, prevented the overproduction of reactive oxygen species, and suppressed the NF-*κ*B inflammasome axis to inhibit PPV-induced apoptosis in PK-15 cells.

## 1. Introduction

Porcine parvovirus (PPV) infection has been acknowledged as a primary cause of reproductive disorders in pregnant cows. It is also a crucial causative agent of diarrhea, skin disease, and arthritis in swine [1, 2]. Clinical pathological changes characteristic of PPV infection include mummification, infertility, embryonic death, stillbirth, and delayed return to estrus [3–5]. PPV is one of the few viruses that is refractory to most disinfectants, and can survive in the environment for extended periods of time. Moreover, no effective treatments are available to improve the outcome of PPV infection in the pig industry, resulting in huge economic losses [6]. Vaccination is an effective form of prevention from PPV replication, but cost and safety issues have been deterrents to routine vaccination in many regions of the world [7]. Therefore, it is necessary to develop new adjunctive drugs to attenuate the effects of PPV infection.

PPV is a single-stranded DNA molecule of approximately 5 kb that contains two major open reading frames (ORFs) and one short, genus-specific ORF. The upstream major ORF encodes two nonstructural proteins (NS1 and NS2) and the downstream major ORF encodes three capsid proteins (VP1, VP2, and VP3) [8–10]. NS1 has helicase and nickase activities and induces apoptosis and cell lysis by participating in viral genome replication, transcriptional regulation, and host cell pathogenicity [11, 12]. Previous studies reported that PPV NS1 leading to DNA and mitochondrial damage induced apoptosis in porcine kidney cells via the endogenous mitochondrial pathway through reactive oxygen species (ROS) accumulation [13]. ROS accumulation was also shown to activate nuclear factor (NF)-*κ*B, resulting in the release of proinflammatory cytokines and cell death [14, 15]. Zhou et al. showed that PPV infection activates inflammatory cytokine production through toll-like receptor (TLR) 9 and NF-*κ*B signaling pathways in porcine kidney cells [16]. Based on this knowledge of the mechanism of PPV, we screened active substances suitable for its targeting.

Compared with vaccines, natural products show low toxicity and high potential clinical translation because of the diversity and complexity of their molecular structure [17]. Such products, including active ingredients of traditional Chinese medicine (TCM), also often exhibit specific high selectivity and biological activities through their ability to regulate multiple signaling pathways [18]. Ferulic acid (FA), a natural product isolate from TCM, is a phenolic compound with antioxidant, anti-inflammatory, antidiabetic, hepatoprotective, and antiviral properties [19–21]. We previously found that FA inhibited PPV replication through the mitochondrial apoptosis pathway. In the present study, we show that FA inhibited PPV-encoded NS1 to contribute to PPV-induced apoptosis as part of the mitochondria-mediated intrinsic apoptosis pathway.

## 2. Methods

### 2.1. Reagents

Monoclonal antibodies against NF-*κ*B, tumor necrosis factor receptor-associated factor (TRAF) 6, I*κ*B kinase (IKK) *α*, MyD88, c-jun N-terminal kinase (JNK), and *β*-actin were purchased from Cell Signaling Technology (Shanghai, China). The DMEM medium and fetal bovine serum (FBS) were from Gibco BRL (Grand Island, NY, USA). The FITC annexin V Apoptosis Detection Kit was from BD Biosciences (San Jose, CA, USA). Lipofectamine™ 2000, dichloro-dihydro-fluorescein diacetate (DCFH-DA), MitoSOX™ Red, MitoTracker Deep Red FM, MitoTracker Green FM, and Hoechst 33342 were from Invitrogen (Carlsbad, CA, USA).

### 2.2. Virus, Cells, and Plasmids

PPV-susceptible PK-15 cells were purchased from the American Type Culture Collection (Gibco BRL) and cultured in DMEM supplemented with 10% heat-inactivated FBS, 100 U/mL penicillin, 100 *μ*g/mL streptomycin, and 2 mM L-glutamine at 37°C in a 5% CO_2_ humidified atmosphere. The PPV SD strain was a generous gift from Professor Zhi Qiang Shen (Shandong Binzhou Animal Science and Veterinary Medicine Academy, Binzhou, China). The pcDNA3.1A plasmid used to construct eukaryotic expression vectors for PPV-encoded genes was purchased from Sangon Biotech Co., Ltd. (Shanghai, China).

### 2.3. Cell Infection and DNA Extraction

PK-15 cells were seeded in 6-well plates at 1 × 10^6^ cells per well and cultured in DMEM complete medium for 24 h. Next, 1000 *μ*L of diluted virus suspension containing a multiplicity of infection (MOI) = 1 of the PPV stock was added to the cells for 4 h. FA at concentrations of 10, 20, or 30 *μ*M was then added to the experimental groups. PPV-infected, mock-treated, and FA-treated cultures were collected at 24 h after infection, and DNA was extracted using the DNAprep pure cell/bacteria kit (TianGen, Beijing, China) according to the manufacturer's instructions.

### 2.4. Quantitative Real-Time Reverse Transcription (RT)-PCR

Primers used for RT-PCR were designed by querying PrimerBank with the Gene ID; sequences are listed in Additional file 1: Table 1. Total RNA was extracted from PK-15 cells using TRIzol reagent (Thermo Fisher Scientific, Shanghai, China) according to the manufacturer's protocol. cDNA of each sample was transcribed by the PrimeScript RT reagent kit (TaKaRa Biotechnology, Beijing, China), and real-time PCR was conducted using SYBR Green I fluorescent dye (TaKaRa Biotechnology, Beijing, China) according to the manufacturer's guidelines.

### 2.5. Western Blot Analysis

PK-15 cells were collected 48 h after PPV infection and lysed in radioimmunoprecipitation assay lysis buffer (50 mM Tris pH 7.4, 150 mM NaCl, 1% Triton X-100, 1% sodium deoxycholate, 0.1% sodium dodecyl sulfate (SDS), sodium orthovanadate, sodium fluoride, EDTA, and leupeptin (Beyotime Institute of Biotechnology, China) to extract total protein. Equal amounts of protein were then subjected to SDS polyacrylamide gel electrophoresis and transferred onto a polyvinylidene fluoride membrane (Millipore, Billerica, MA). After blocking with Tris-buffered saline with Tween 20 containing 5% nonfat dried milk (Becton, Dickinson and Company, Franklin Lakes, NJ, USA), the membranes were incubated with retinoic acid receptor beta polyclonal primary antibody (ProteinTech, Beijing, China) and corresponding horseradish peroxidase-conjugated secondary antibodies (ProteinTech). Protein bands were detected by enhanced chemiluminescence (Millipore, Billerica, MA).

### 2.6. Construction of PPV NS1 Expression Vectors

NS1 genes were PCR-amplified from the PPV genome using specific primers (Table 1) according to a previously described vector construction protocol [13].

### 2.7. PPV Infection and NS1 Expression Vector Transfection

PK-15 cells were plated at 1 × 10^6^ per well in 6-well plates and cultured in DMEM complete medium for 24 h and then infected with PPV at a MOI of 1. After a further 24 h, the DMEM medium was removed. NS1 vector (4 *μ*g/*μ*L) and 10 *μ*L Lipofectamine 3000 reagent (Beyotime Biotechnology, Shanghai, China) were separately mixed with 250 *μ*L of the DMEM complete medium, allowed to stand at room temperature for 5 min, and then lightly mixed together and added to the cells for 20 min of culture at room temperature. Cells were then incubated with 500 *μ*L mixed medium for 8 h at 37°C and recultured in complete medium for an additional 16 h at 37°C. Luciferase activities were detected using a TD-20/20 luminometer (Turner BioSystems, Inc., Sunnyvale, CA, USA). The pcDNA3.1A vector was transfected as mentioned above as a negative control, and different concentrations of FA were applied to the experimental groups.

### 2.8. Cell Viability Assay

PK-15 cell viability was evaluated by using the CCK-8 kit (Beyotime Biotechnology) following the manufacturer's instructions.

### 2.9. Statistical Analysis

Results were analyzed by one-way analysis of variance and the Student's *t*-test with Bonferroni correction. All numerical data were collected from at least three separate experiments. Results were expressed as means ± standard deviation of the means. *P* < 0.05 was considered statistically significant.

## 3. Results

### 3.1. FA Inhibition of PPV-Induced Apoptosis Mainly Occurred through NS1 Protein

**Figure 1:**
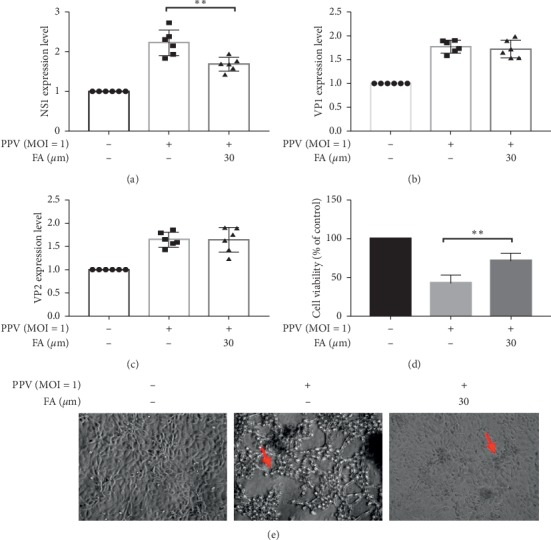
Inhibition of PPV infection-induced apoptosis via NS1. PPV (MOI = 1) was used to infect PK-15 cells with or without FA treatment, and cell supernatants were harvested to determine the expression of NS1 (a), VP1 (b), and VP2 (c) by RT-PCR. Cell viability was detected by using the CCK-8 kit (d). ^*∗∗*^*P* < 0.01 compared with the PPV but no-FA group. Cell cytopathic changes were observed microscopically (e), and red arrows were cell cytopathic changes.

PPV infection is known to induce apoptosis in PK-15 cells, and we previously found that this could be inhibited by FA [1, 2]. To further investigate the mechanism by which FA inhibits PPV-induced apoptosis of PK-15 cells, we examined the expression of PPV-encoded genes by RT-PCR (Figure 1(a)). PPV infection significantly upregulated the expression of NS1 compared with the mock group, and this was significantly downregulated by treatment with FA. However, FA had no significant effect on the PPV-induced expression of VP1 or VP2 (Figures 1(b) and 1(c)). As shown in Figure 1(d), FA significantly inhibited PPV-induced apoptosis of PK-15 cells. Together, these data indicate that NS1 protein plays an important role in the inhibition of PPV-induced apoptosis by FA.

### 3.2. FA Inhibition of Inflammatory Cytokine Production after PPV Infection

**Figure 2:**
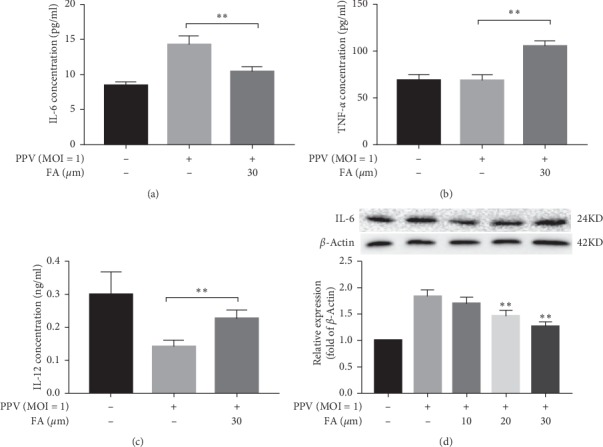
Inhibition of PPV infection-induced IL-6, TNF-*α*, and IL-12 expression in PK-15 cells. PPV (MOI = 1) was used to infect PK-15 cells with or without FA treatment, and cell culture supernatants were harvested to determine the concentration of IL-6 ((a) and (d)), TNF-*α* (b), and IL-12 (c) using ELISA kits and western blotting. ^*∗∗*^*P* < 0.01 compared with the PPV but no-FA group.

Viral infection could induce the release of inflammatory cytokines. To determine whether FA would inhibit inflammatory cytokine production in PK-15 cells after PPV infection, cell culture supernatants were harvested and used to detect interleukin (IL)-6, IL-12, and tumor necrosis factor (TNF)-*α* by ELISA (Boster Biotechnology, Wuhan, China) following the manufacturer's protocol. PPV infection was found to stimulate IL-6 secretion and inhibit TNF-*α* and IL-12 secretion (Figures 2(a)–2(c)), but significant downregulation of IL-6 secretion was observed following FA treatment. This was further verified by determining IL-6 mRNA and protein expression using quantitative real-time PCR (Figure 2(d)) and western blotting.

### 3.3. FA Inhibition of PPV-Induced Apoptosis Involved NF-κB Signaling Pathway-Related Genes

**Figure 3:**
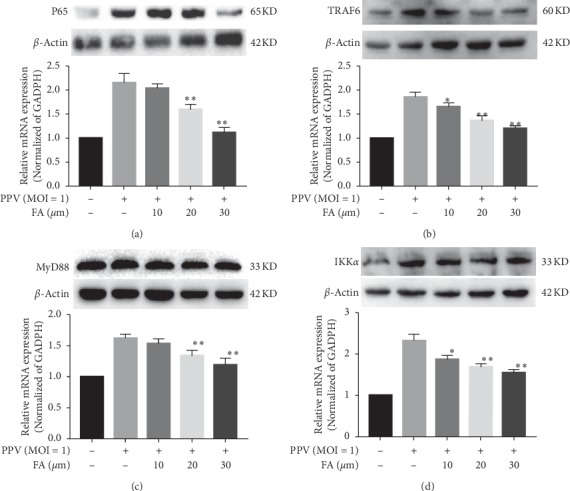
Inhibition of PPV infection-induced transcription of NF-*κ*B pathway-related genes in PK-15 cells. The mRNA and protein expression levels of P65 (a), TRAF6 (b), MyD88 (c), and IKK*α* (d) were detected by RT-PCR and western blotting. ^*∗∗*^*P* < 0.01 and ^*∗*^*P* < 0.05 compared with the PPV but no-FA group. All data are expressed as the mean ± SD of three independent experiments.

FA was previously shown to suppress excessive ROS production, NF-*κ*B/NLRP3 inflammasome axis activation, and apoptosis [16]. To determine the expression profile of NF-*κ*B signaling pathway-related genes in PK-15 cells, mRNA and protein expression levels of NF-*κ*B, TRAF6, IKK*α*, and MyD88 were examined using quantitative real-time PCR and western blotting. PPV infection significantly increased both the gene and protein expression levels of NF-*κ*B, TRAF6, IKK*α*, and MyD88 compared with the uninfected group. However, FA treatment significantly suppressed this increase in a dose-dependent manner (Figures 3(a)–3(d)).

### 3.4. FA Inhibition of PPV-Induced Apoptosis Involved NF-*κ*B Signaling Pathways Mediated by TLR4

**Figure 4:**
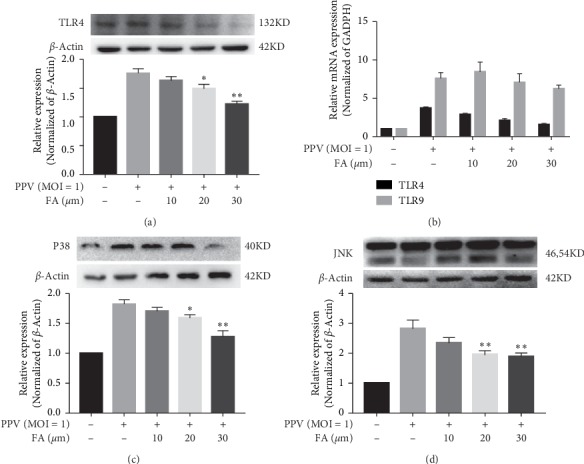
PPV bound to TLR4 and suppressed MAPK signaling pathway-related genes. TLR4 and TLR9 mRNA expression levels were detected by RT-PCR ((a), (b)). TLR4 protein expression was measured by western blotting (a). MAPK signaling pathway-related genes ((c), (d)) were analyzed by RT-PCR and western blotting. All data are expressed as the mean ± SD of three independent experiments. ^*∗*^*P* < 0.05 and ^*∗*^*P* < 0.05 compared with the PPV but no-FA group.

TLRs are the first line of defense against invading pathogens, and are important pattern recognition receptors for the detection and response of microbial ligands upstream of the NF-*κ*B pathway [16]. Early PPV infection was previously shown to activate TLR1–TLR10. To determine whether TLRs play key roles in identifying the virus during FA inhibition of PPV-induced apoptosis, we used RT-PCR and western blotting to investigate the transcription pattern of TLR1–10. As shown in Figures 4(a) and 4(b), PPV could upregulate the gene and protein expression levels of TLR4 and TLR9 compared with the uninfected group. FA significantly downregulated the expression of TLR4; although the same effect was seen on TLR9, 20 and 30 *μ*M FA groups could downregulate the expression of TLR9 compared with the uninfected group. These results indicated that FA inhibited PPV-induced apoptosis via TLR4.

Mitogen-activated kinases (MAPKs) including extracellular signal-regulated JNK and p38 MAPK are important signaling molecules following TLR4 activation [22]. As shown in Figures 4(c) and 4(d), gene and protein expression levels of P38 MAPK and JNK were significantly upregulated in response to PPV infection, but this was inhibited by FA treatment. Together, these data suggest that FA inhibited PPV-induced apoptosis in PK-15 cells through the NF-*κ*B signaling pathway mediated by TLR4.

### 3.5. FA Inhibition of NS1 Protein-Activated NF-*κ*B Signaling Pathways and ROS Accumulation

**Figure 5:**
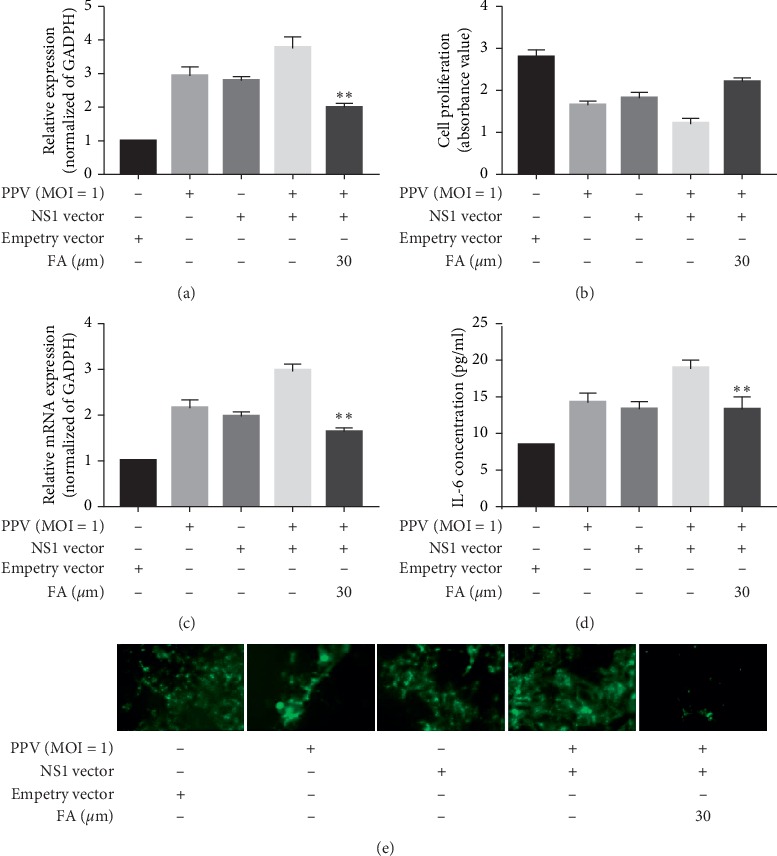
Inhibition of NS1 protein-activated NF-*κ*B signaling pathways and ROS accumulation. NS1 expression following NS1 vector transfection and FA treatment (a). Cell survival rates under NS1 vector transfection and FA treatment (b). NF-*κ*B (c) and IL-6 (d) expression under NS1 vector transfection and FA treatment. ROS expression following NS1 vector transfection and FA treatment as detected by DCFH-DA (e).

NS1 plays an important role in PPV infection of host cells. Previous work showed that PPV infection actives NF-*κ*B signaling pathways and induces ROS accumulation, thereby inducing apoptosis in host cells [16, 23]. However, it was unclear whether this involved the NS1 protein. PK-15 cells were transfected with NS1 vector (4 *μ*g) and underwent FA treatment, and then NS1 and NF-*κ*B expression was analyzed by RT-PCR and intracellular ROS levels were determined by the DCFH-DA fluorescence assay. As shown in Figures 5(a) and 5(b), the relative expression of NS1 in cells infected with PPV, transfected with the NS1 vector, and receiving FA treatment was significantly lower than that of cells not treated with FA; moreover, cell proliferation was notably higher. This indicated that FA treatment significantly inhibited PPV and NS1 vector-induced apoptosis in PK-15 cells. FA treatment also significantly downregulated the expression of NF-*κ*B (Figure 5(c)) and IL-6 (Figure 5(d)) induced by PPV and NS1 vector coinfection and reduced the expression of ROS (Figure 5(e)). These data indicate that FA inhibited NS1 protein-activated NF-*κ*B signaling pathways and ROS accumulation.

## 4. Discussion

FA is known to have pharmacological bioactivity, including radioresistance, antioxidant, antibacterial, and antiviral functions [24]. Our previous studies showed that FA could inhibit PPV infection both *in vitro* and *in vivo*, but the mechanism of action of this was unclear. In the present study, we revealed that FA interference of PPV NS1 protein expression inhibited PPV-induced apoptosis in PK-15 cells mainly via TLR4 and NF-*κ*B signaling pathways.

Virus infection-induced apoptosis plays an important role in viral pathogenesis. The PPV NS1 protein mainly induces apoptosis via the ROS/mitochondrial pathway [13], and ROS-mediated NF-*κ*B activation and subsequent upregulation of inducible nitric oxide (NO) synthase increase NO levels. Such increases in ROS and NO can lead to DNA and protein damage, resulting in cell death [20]. At different phases of the virus life cycle, viral infection affects NF-*κ*B signaling [25]. Indeed, the activation of NF-*κ*B signaling by PPV infection is well-documented [16]. In the present study, the expression of ROS and NF-κB in PK-15 cells infected with PPV was increased. FA confers protection against ROS-induced mitochondrial dysfunction and NF-*κ*B signaling-induced apoptosis, so suppressing NF-*κ*B signaling and enhancing cellular antioxidant defenses was predicted to prevent PPV infection-induced apoptosis. As expected, treatment with FA could suppress PPV infection-induced NF-*κ*B signaling-related genes and the production of ROS.

The activation of inflammation has previously been implicated in the development of PPV infection-induced apoptosis [13, 14, 16]. In the present study, PPV infection-induced apoptosis by activating the expression of P53 and enhancing ROS, which is known to activate redox-sensitive NF-*κ*B and its downstream inflammatory mediators. Additionally, ROS integrate different signals leading to inflammasome activation [26]. PPV- and NS1-induced ROS accumulation therefore appears to be a crucial factor for PPV infection-induced apoptosis in PK-15 cells. In our study, PPV infection-induced ROS overproduction was also associated with the activation of NF-*κ*B, together with increased secretion of IL-6. The prevention of PPV-induced NF-*κ*B activation and ROS accumulation by FA subsequently ameliorated IL-6 levels.

To further verify the mechanisms underlying the ameliorative effect of FA on PPV-induced apoptosis, we determined the expression levels of TLRs. These are key initiators of innate immune responses to macrophage infection and to cells in the adaptive immune system. TLR4 identifies exogenous pathogens by binding to lipopolysaccharides of Gram-negative bacteria, stimulating the production of antimicrobial peptides, and inducing nonspecific immune responses such as activation of the NF-*κ*B pathway in macrophages. FA was previously documented to prevent apoptotic cell death by suppressing oxidative stress and the expression of Bax, TLR4, and caspase-3 genes [27]. Based on our findings, we propose that FA inhibits NF-*κ*B activation by a downstream mechanism involving TLR4.

In conclusion, PPV infection activated the expression of TLR4, the NF-*κ*B inflammasome axis, and its downstream molecule IL-6 in PK-15 cells. FA markedly prevented the overproduction of ROS, inflammation, and apoptosis in PPV-infected cells and enhanced their antioxidant defenses. TLR4 was found to underlie these ameliorative effects involving the suppression of ROS and NF-*κ*B inflammasome signaling, as shown by the mechanistic pathways. Thus, FA has potential as a promising protective agent against PPV infection-induced apoptosis. However, further testing should be conducted *in vivo*.

## Figures and Tables

**Table 1:** Primers for amplifying porcine parvovirus-encoded genes.

Gene	Primer name	Primer sequence (5'⟶3')	Size (bp)
NS1	NS1F	CGGGGTACCACCATGGCAGCGGGAAACACTTAC	2007
	NS1R	CCGACCGGTTTCAAGGTTTGTTGTGGGTGC	

VP1	VP1F	CGGGGTACCACCATGGCGCCTCCTGCAAAAAGAGCA	2179
	VP1R	CCGACCGGTGTATAATTTTCTTGGTATAAGTTG	

VP2	VP2F	CGGGGTACCACCATGAGTGAAAATGTGGAACAAC	1758
	VP2R	CCGACCGGTGTATAATTTTCTTGGTATAAGTTG	

NF-*κ*B	NF-*κ*BF	GCAGCAAGCAGAAGAGCA	145
	NF-*κ*BR	CAGCCCACAGCAACAGAG	

TRAF6	TRAF6F	AGGGAACGATACGCCTTAC	118
	TRAF6R	CGTGGGATTGTGGGTCT	

MYD88	MYD88F	CGTCGGATGGTAGTGGTTG	100
	MYD88R	TCTGATGGGCACCTGGA	

IL-6	IL-6F	CTCAGCAATGTGGGCTGT	101
	IL-6R	TCTTCCACGGGACTGTTCT	

TLR4	TLR4F	GCCTCCAAACCTTGAAAA	139
	TLR4R	GAATGAAATGCCCTCTGG	

## Data Availability

All data generated or analyzed during this study are included in this published article and its supplementary information files.

## Supplementary Materials

Supplementary Materials TRAF6 *β*-actin uninfected group, 2. PPV-infected, 3. 10 *μ*m FA-treated, 4. 20 *μ*m FA-treated, 30 *μ*m FA-treated TRAF6 uninfected group, 2. PPV-infected, 3. 10 *μ*m FA-treated, 4. 20 *μ*m FA-treated, 30 *μ*m FA-treated.

## References

[1] Gava D., Souza C. K., Schaefer R. (2015). A TaqMan-based real-time PCR for detection and quantification of porcine parvovirus 4. *Journal of Virological Methods*.

[2] Chen H.-Y., Li X.-K., Cui B.-A. (2009). A TaqMan-based real-time polymerase chain reaction for the detection of porcine parvovirus. *Journal of Virological Methods*.

[3] Song C., Zhu C., Zhang C., Cui S. (2010). Detection of porcine parvovirus using a taqman-based real-time pcr with primers and probe designed for the NS1 gene. *Virology Journal*.

[4] Hueffer K., Parrish C. R. (2003). Parvovirus host range, cell tropism and evolution. *Current Opinion in Microbiology*.

[5] Johnson R., Collings D. (1969). Experimental infection of piglets and pregnant gilts with a parvovirus. *Veterinary Record*.

[6] Opriessnig T., Fenaux M., Yu S. (2004). Effect of porcine parvovirus vaccination on the development of PMWS in segregated early weaned pigs coinfected with type 2 porcine circovirus and porcine parvovirus. *Veterinary Microbiology*.

[7] Li P., Zou H., Ren Y., Zarlenga D. S., Ren X. (2014). Antiviral effect of diammonium glycyrrhizinate on cell infection by porcine parvovirus. *Current Microbiology*.

[8] Mészáros I., Tóth R., Olasz F., Tijssen P., Zádori Z. (2017). The SAT protein of porcine parvovirus accelerates viral spreading through induction of irreversible endoplasmic reticulum stress. *Journal of Virology*.

[9] Ji P., Liu Y., Chen Y. (2017). Porcine parvovirus capsid protein expressed in *Escherichia coli* self-assembles into virus-like particles with high immunogenicity in mice and guinea pigs. *Antiviral Research*.

[10] Fernandes S., Boisvert M., Szelei J., Tijssen P. (2014). Differential replication of two porcine parvovirus strains in bovine cell lines ensues from initial DNA processing and NS1 expression. *Journal of General Virology*.

[11] Daeffler L., Hörlein R., Rommelaere J., Nüesch J. P. F. (2003). Modulation of minute virus of mice cytotoxic activities through site-directed mutagenesis within the NS coding region. *Journal of Virology*.

[12] Sol N., Junter J. L., Vassias I. (1999). Possible interactions between the NS-1 protein and tumor necrosis factor alpha pathways in erythroid cell apoptosis induced by human parvovirus B19. *Journal of Virology*.

[13] Zhang J., Fan J., Li Y. (2019). Porcine parvovirus infection causes pig placenta tissue damage involving nonstructural protein 1 (NS1)-induced intrinsic ROS/mitochondria-mediated apoptosis. *Viruses*.

[14] Loverre A., Ditonno P., Crovace A. (2004). Ischemia-reperfusion induces glomerular and tubular activation of proinflammatory and antiapoptotic pathways: differential modulation by rapamycin. *Journal of the American Society of Nephrology*.

[15] Fink S. L., Cookson B. T. (2005). Apoptosis, pyroptosis, and necrosis: mechanistic description of dead and dying eukaryotic cells. *Infection and Immunity*.

[16] Zhou Y., Jin X.-h., Jing Y.-x. (2017). Porcine parvovirus infection activates inflammatory cytokine production through toll-like receptor 9 and NF-*κ*B signaling pathways in porcine kidney cells. *Veterinary Microbiology*.

[17] Li X., Lee S., Yoon J. (2018). Supramolecular photosensitizers rejuvenate photodynamic therapy. *Chemical Society Reviews*.

[18] Xiao Q., Wu J., Pang X. (2018). Discovery and development of natural products and their derivatives as photosensitizers for photodynamic therapy. *Current Medicinal Chemistry*.

[19] Perez-Ternero C., Werner C. M., Nickel A. G. (2017). Ferulic acid, a bioactive component of rice bran, improves oxidative stress and mitochondrial biogenesis and dynamics in mice and in human mononuclear cells. *The Journal of Nutritional Biochemistry*.

[20] Narasimhan A., Chinnaiyan M., Karundevi B. (2015). Ferulic acid exerts its antidiabetic effect by modulating insulin-signalling molecules in the liver of high-fat diet and fructose-induced type-2 diabetic adult male rat. *Applied Physiology, Nutrition, and Metabolism*.

[21] Yuan J., Ge K., Mu J. (2016). Ferulic acid attenuated acetaminophen-induced hepatotoxicity though down-regulating the cytochrome P 2E1 and inhibiting toll-like receptor 4 signaling-mediated inflammation in mice. *American Journal of Translational Research*.

[22] Kyriakis J. M., Avruch J. (2001). Mammalian mitogen-activated protein kinase signal transduction pathways activated by stress and inflammation. *Physiological Reviews*.

[23] Zhao X., Xiang H., Bai X. (2016). Porcine parvovirus infection activates mitochondria-mediated apoptotic signaling pathway by inducing ROS accumulation. *Virology Journal*.

[24] Hirabayashi T., Ochiai H., Sakai S., Nakajima K., Terasawa K. (1995). Inhibitory effect of ferulic acid and isoferulic acid on murine interleukin-8 production in response to influenza virus infectionsin vitroandin vivo. *Planta Medica*.

[25] de Oliveira D. E., Ballon G., Cesarman E. (2010). NF-*κ*B signaling modulation by EBV and KSHV. *Trends in Microbiology*.

[26] Tschopp J., Schroder K. (2010). NLRP3 inflammasome activation: the convergence of multiple signalling pathways on ROS production?. *Nature Reviews Immunology*.

[27] Ren Z., Zhang R., Li Y., Li Y., Yang Z., Yang H. (2017). Ferulic acid exerts neuroprotective effects against cerebral ischemia/reperfusion-induced injury via antioxidant and anti-apoptotic mechanisms in vitro and in vivo. *International Journal of Molecular Medicine*.

