# Supplementary material for: Ferulic Acid Protects against Porcine Parvovirus Infection-Induced Apoptosis by Suppressing the Nuclear Factor-κB Inflammasome Axis and Toll-Like Receptor 4 via Nonstructural Protein 1
Source: Evid Based Complement Alternat Med. 2020 Apr 25;2020:3943672. doi: 10.1155/2020/3943672 (PMC7199543; doi:10.1155/2020/3943672)
Supplement: Supplementary Materials — TRAF6 β-actin uninfected group, 2. PPV-infected, 3. 10 μm FA-treated, 4. 20 μm FA-treated, 30 μm FA-treated TRAF6 uninfected group, 2. PPV-infected, 3. 10 μm FA-treated, 4. 20 μm FA-treated, 30 μm FA-treated. [file 3943672.f1.docx]

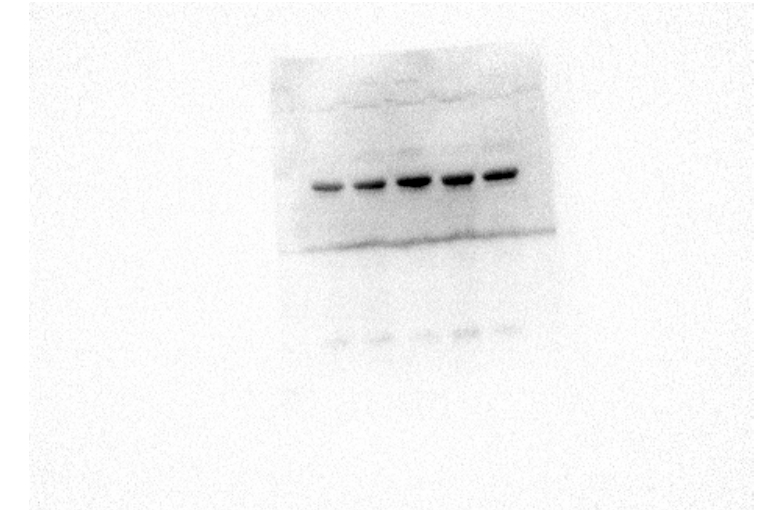


42KD

**1 2 3 4 5**

TRAF6 β-Actin

1. Uninfected group, 2. PPV-infected, 3. 10μm FA-treated, 4. 20μm FA-treated, 30μm FA-treated


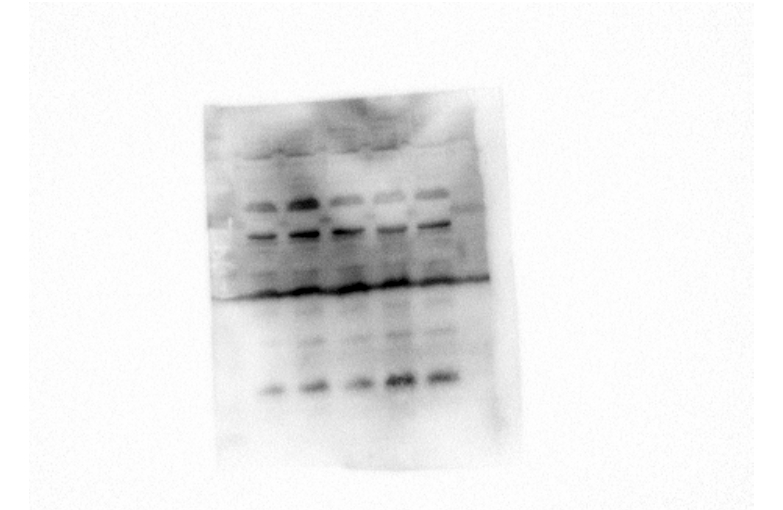


TRAF6

1. Uninfected group, 2. PPV-infected, 3. 10μm FA-treated, 4. 20μm FA-treated, 30μm FA-treated

The supplement of original data

60KD

**1 2 3 4 5**
